# Effects of two flap palatoplasty versus furlow palatoplasty with buccal myomucosal flap on maxillary arch dimensions in patients with cleft palate at the primary dentition stage: a cohort study

**DOI:** 10.1007/s00784-023-05182-0

**Published:** 2023-08-02

**Authors:** Mamdouh Ahmed Aboulhassan, Shaimaa Mohsen Refahee, Shaimaa Sabry, Mohamed Abd-El-Ghafour

**Affiliations:** 1 Plastic Section, Department of General Surgery, Faculty of Medicine, Cairo University, Cairo, 11111 Egypt; 2 Oral and Maxillofacial Surgery Department, Faculty of Dentistry, Fayoum University, Fayoum, 63511 Egypt; 3 Department of Pediatric Dentistry and Public Health, Faculty of Dentistry, Cairo University, Cairo, 11111 Egypt; 4 Department of Orthodontics, Faculty of Dentistry, Cairo University, Cairo, 11111 Egypt

**Keywords:** Cleft palate, Furlow with buccal myomucosal flap palatoplasty, Two flap palatoplasty, Maxillary dental arch

## Abstract

**Objective:**

The objective of this study was to evaluate the effect of two flap palatoplasty (TFP) versus Furlow palatoplasty with buccal myomucosal flap (FPBF) on maxillary arch dimensions in children at the primary dentition stage with cleft palate, in comparison to matching subjects without any craniofacial anomalies.

**Material and methods:**

This study included 28 subjects with an age range of 5–6 years; 10 non-cleft subjects were included in the control group, 9 patients treated with TFP, and 9 patients treated with FPBF. For the included patients, the maxillary models were scanned using a desktop scanner to produce virtual models, and the maxillary dimension measurements were virtually completed. The produced measurements were compared between the 3 groups. Maxillary models of the 28 participants were evaluated.

**Results:**

Statistically insignificant differences were detected between the 3 groups for arch symmetry measurements. Differences were detected in the inter-canine width between the 2 surgical groups and non-cleft group. Both arch length and posterior palatal depth significantly differ while comparing the TFP to the control group, with no differences between FPBF and the non-cleft group.

**Conclusion:**

Furlow palatoplasty with buccal myomucosal flap might be considered a better surgical option than two flap palatoplasty for patients with cleft palate while evaluating maxillary arch dimensions at the primary dentition stage as a surgical outcome.

**Clinical relevance:**

This study gives insight into the surgical technique that has limited effect on the maxillary growth and dental arch dimension. Therefore, it decreases the need for orthodontic treatment and orthognathic surgery.

**Trial registration:**

clinicaltrials.gov (NCT05405738).

## Introduction

Cleft lip and palate are considered the most common craniofacial anomaly [1]. Speech development, velopharyngeal function enhancement, improvement of Eustachian tube function, and maxillofacial growth are the primary goals of cleft palate repair [2–6]. Early cleft palate reconstruction is needed to reach these goals, especially restoring speech and improving Eustachian tube dysfunction to minimize middle ear infections [7, 8].

An unavoidable side effect of palatal surgical repair is the development of the palatal scar with its potential impairing effect on the growth of the maxillofacial structures. About 25 to 60% of cleft patients experienced maxillary hypoplasia in transverse, sagittal, and vertical dimensions after cleft repair [9, 10]. Additionally, 70% of the patients have skeletal class 3 which occurs due to scar contracture at the surgical site [11, 12]. Generali C [13] et al. in 2017, evaluated the dental casts of unilateral cleft patients and concluded that unilateral cleft is associated with the narrow maxilla and high vault palate that led to skeletal class 3, crossbite, anterior open bite, dental crowding, and mouth breathing.

Different studies suggested that maxillary growth is affected by the patient’s age, the timing of repair, surgical technique, treatment protocol, and surgeon skills [13–17]. However, Corthouts P [18] et al. in 2020 concluded that surgical technique is the main factor that affects maxillary growth.

Various surgical techniques are followed for cleft palate repair, such as von Langenbeck’s bipedicle flap technique, Veau-Wardill-Kilner pushback technique, Bardach’s two-flap palatoplasty (TFP), Furlow double opposing Z-Plasty, and Furlow palatoplasty with buccal myomucosal flap (FPBF) [19–21]. Two flap palatoplasty is considered the most commonly used technique due to its low rate of fistula but it is the most harmful technique causing maxillary growth restriction [22–26]. Rossell-Perry et al. [10] approved that there was no significance regarding maxillary growth and dental arch dimension between one flap and two flap techniques. In addition, the maxillary growth restriction is due to scar formation of relaxing incision, not related to the amount of exposed hard palate bone only.

Nowadays, a lot of centers shifted the surgical cleft palate repair to FPBF due to its advantages. Furlow palatoplasty with buccal myomucosal flap allows tension-free closure even with a wide cleft. It decreases the scar burden which may affect the growth of the maxilla and dental arch dimension [26, 27].

Although a lot of studies evaluate the effect of different surgical techniques on maxillofacial growth, none of them compare the effect of two flap palatoplasty techniques and Furlow palatoplasty with buccal myomucosal flap on the maxillary development pattern of the cleft patient.

Accordingly, the objective of the current study was to evaluate the effect of two flap palatoplasty versus Furlow palatoplasty with buccal myomucosal flap on dental arch dimensions in children at the primary dentition stage with cleft palate, in comparison to matching normal subjects without any craniofacial anomalies.

## Methods

### Study design

This cohort study was approved by the University Supreme Committee for Scientific Research Ethics (EC2204). The study protocol followed Helsinki Declaration’s statement, Strengthening the Reporting of Observational studies in Epidemiology (STROBE) checklist [28, 29], and registered on clinicaltrials.gov (NCT05405738).

### Setting

All the impressions were made in the outpatient clinic, Dentistry School from May 2022 to March 2023. All patients’ guardians documented their approval to participate in the trial and signed the informed consent.

### Participants

This study included 28 medically free female patients with an age range of 5–6 years. Regarding the cleft groups, patients exposed to secondary cleft lip or palate repair, wound dehiscence, or palatal fistula were excluded. The participating patients must be with non-syndromic complete cleft palate, and all treated with the same surgeon. All the cleft palate defects were repaired at the age of 9–12 months either by TFP or FPBF.

The included groups are the control group including 10 non-cleft subjects, the TFP group including 9 patients treated with TFP, and the FPBF group including 9 patients treated with FPBF.

### The surgical techniques

All surgeries were done by a single experienced plastic pediatric surgeon.

#### Two flap palatoplasty (TFP): [30] (Fig. 1a, b)

After adrenaline 1/200,000 with local anesthesia at the surgical site, the lateral incision was made medial to the teeth from the incisive foramen to the Hamulus, and the medial incision was made around the cleft edge. The palatal mucoperiosteum was elevated and dissected from the underlying bone. Freeing the velar muscles from its abnormal attachment to the posterior border of the hard palate. The nasal layer was closed either directly or with the use of a vomerine flap in wide cases followed by the muscular layer. Finally, the oral mucosa was sutured at the midline area to close the cleft in three layers.

**Fig. 1:**
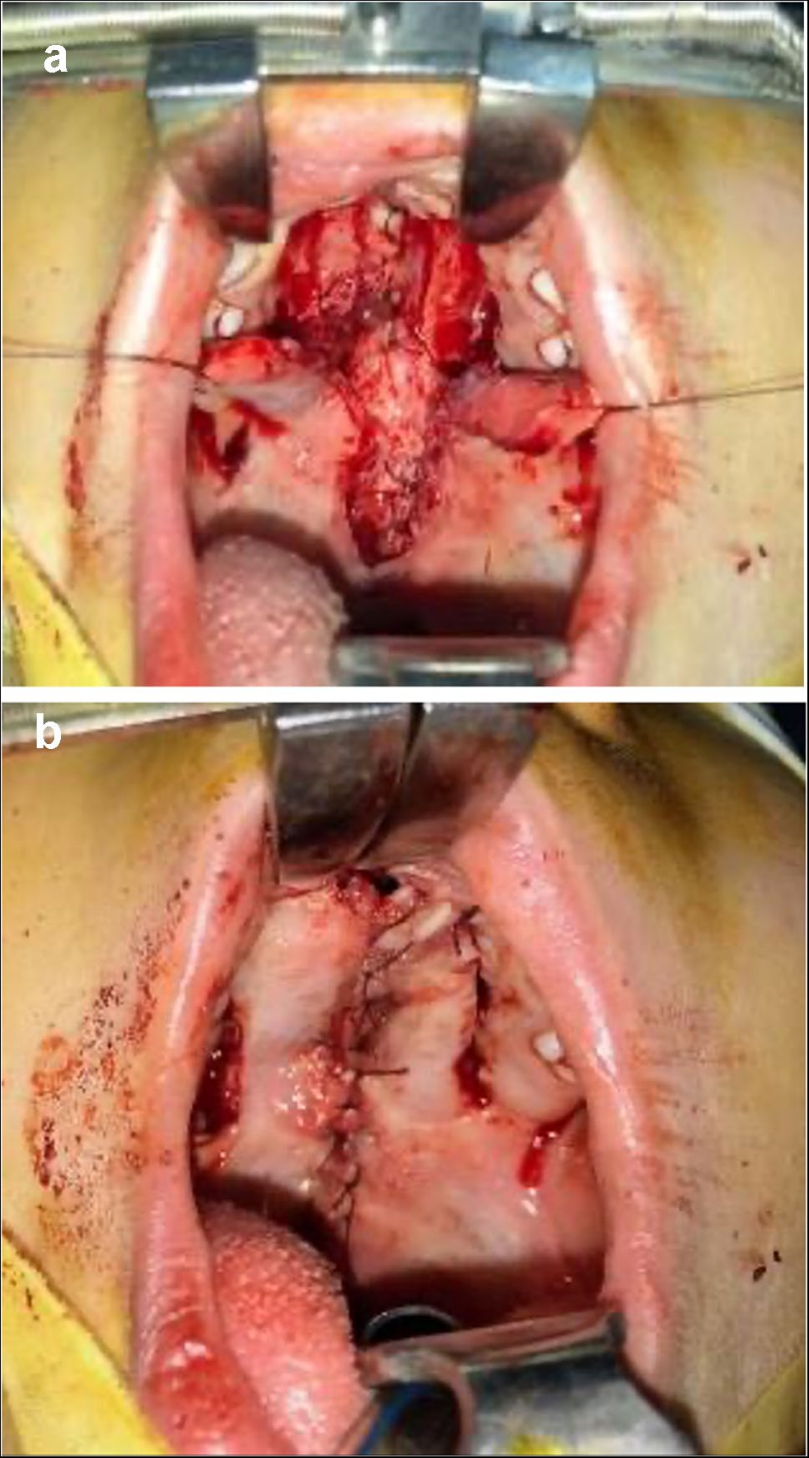
Two flap palatoplasty surgical technique. **a** Showed reflection of the oral mucosa and nasal mucosal layer closure in two flap palatoplasty surgical technique; **b** Showed immediate postoperative view of the operated cleft by two flap palatoplasty technique

#### Furlow palatoplasty with buccal myomucosal flap (FPBF): [21] (Fig. 2a, b, c, d)

After marking of the flap and BMMF outline with methelyne blue, a right side anteriorly based oral mucoperiosteal flap with an angle of about 90° and a left side posteriorly based oral mucoperiosteal flap with an angle of about 60° containing the palatal muscle were incised and elevated. This is followed by disinsertion of the right palatal muscles from the bony hard palate and incising of the nasal mucosa a few mm distal to the bone thus having a posteriorly passed nasal myomucosal flap. On the left side, an anteriorly based nasal myomucosal flap was created by 60° angular incision. Closure of the nasal layer from the uvula to the hard palate using these alternating flaps without overdue tension. Anteriorly nasal layer closure was continued either directly or by using a vomerian flap. Posteriorly, the flaps are closed without overdue tension so that the muscle would only cross the middle line for a few millimeters. Finally, the oral layer was closed with minimal overlapping to the muscle and the residual defect was closed by myomucosal flap ensuring the retro-positioning of the Z plasty repair.

**Fig. 2:**
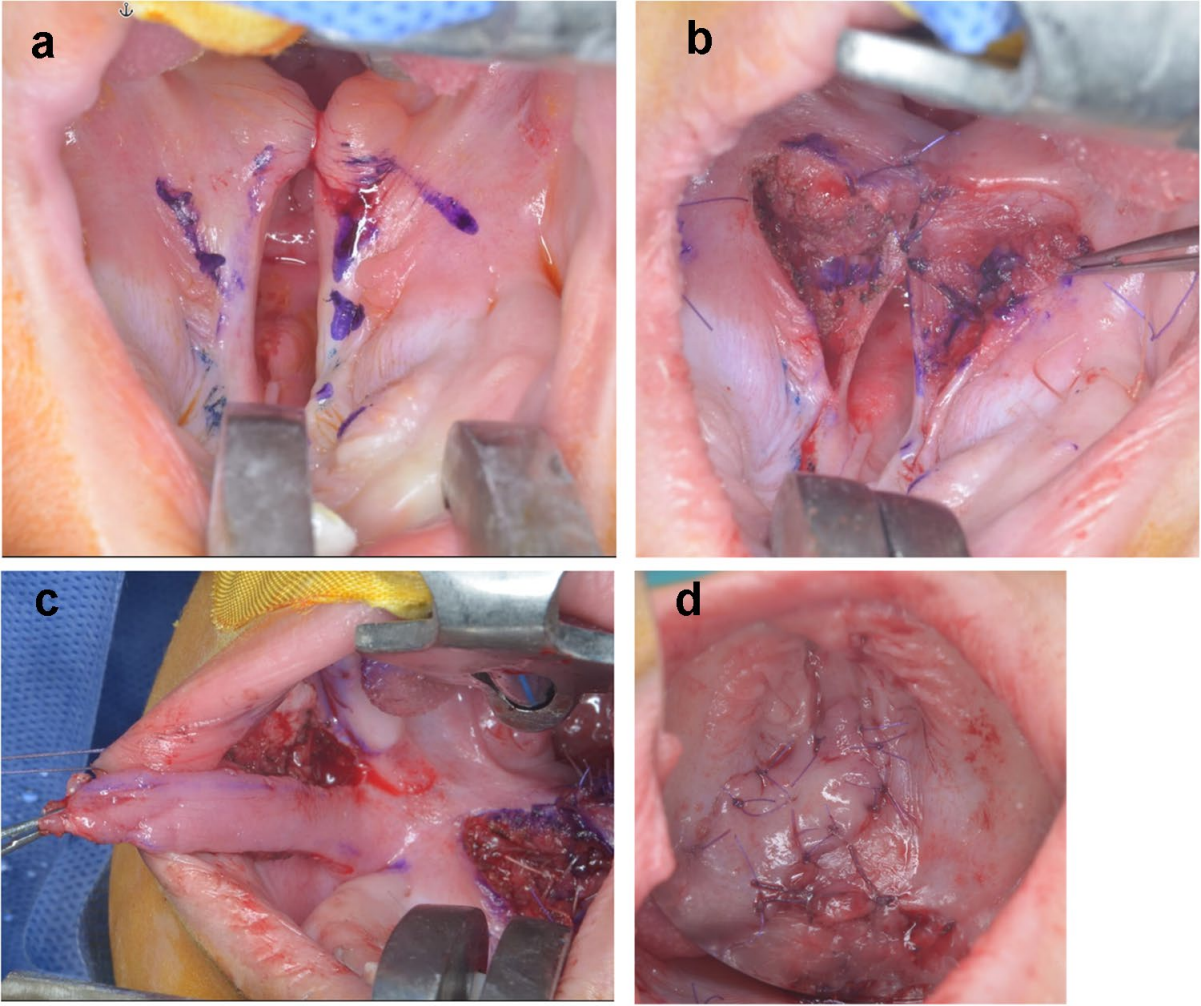
Furlow palatoplasty with buccal myomucosal flap surgical technique. **a** Showed preoperative view with Furlow palatoplasty incision outline (Z-plasty on the oral side and the nasal mucosa); **b** nasal mucosal layer closure; **c** showed the buccal flap incision; **d** showed the immediate postoperative view after buccal flap rotation and closure of the palate residual gap between the soft and hard palate on the oral mucosal side

### Data sources/measurement

For all the included patients, alginate impressions were made at the age of 5–6 years. After 15 min, the impressions were poured with extra hard stone and trimmed to be with a base of 5 mm height. All the models were scanned using the desktop scanner (3shape Lab Scanner- R500) and software (3shape Scan-it Manager ^TM^) aiming to produce the virtual models.

**Fig. 3:**
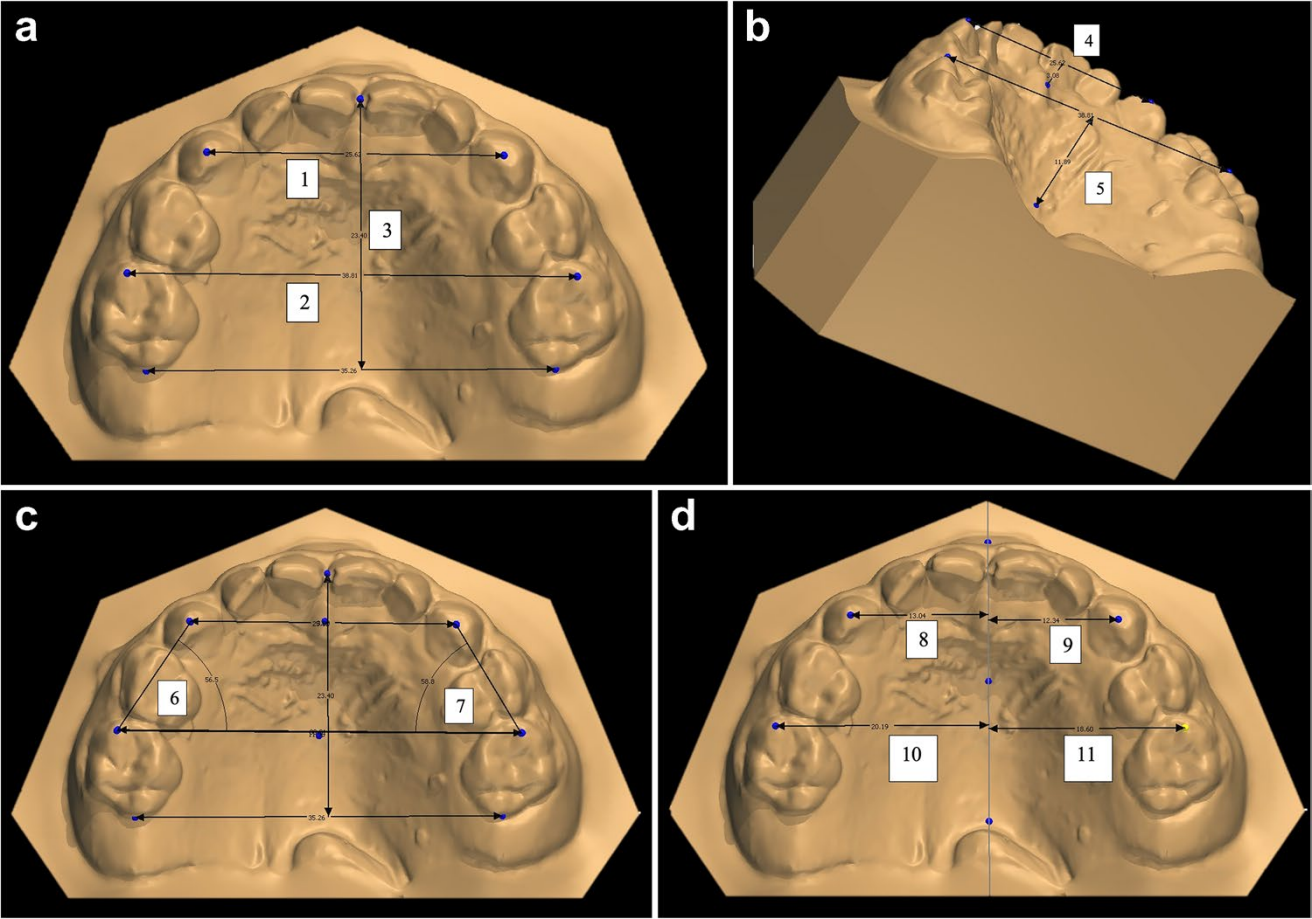
**a** Model’s measurements; 1- Inter-canine width (mm), 2- Intermolar width (mm), 3- Arch Length (mm), **b** Model’s measurments;4- Anterior Palatal Depth (mm), 5-Posterior Palatal Depth (mm), **c** Model’s measurments; 6- Right Side Angle (degrees), 7- Left Side Angle (degrees), **d** Model’s measurements; 8- Right Canine Distance to midline (mm), 9- Left Canine Distance to midline (mm), 10- Right Molar Distance to midline (mm), 11- Left Molar Distance to midline (mm)

Using desktop software (3shape Ortho Analyzer ^TM^), the maxillary dimension measurements were completed. Eleven measurements were done for each model in all the 3 groups. All the measurements are mentioned in Table 1 and Figure 3 [31].

**Table 1:** The used measurements and definitions

Measurements		Definition
1	**Inter-canine width (mm)**	Distance measured between right and left deciduous maxillary canines cusp tips.
2	**Intermolar width (mm)**	Distance measured between right and left deciduous maxillary second molars mesiobuccal cusp tips.
3	**Arch length (mm)**	Distance between the contact point of the maxillary deciduous central incisors to the corresponding point on a line connecting the most distal point of the right and left deciduous maxillary second molars.
4	**Anterior palatal depth (mm)**	Distance between the line connecting the right and left deciduous maxillary canine cusp tips and the deepest point the palatal vault at that line.
5	**Posterior palatal depth (mm)**	Distance between the line connecting the right and left deciduous maxillary second molars mesiobuccal cusp tips and the deepest point the palatal vault at that line.
Arch symmetry measurements		
6	**Right side angle (degrees)**	The angle between the right deciduous canine cusp tip to the right deciduous second molar mesiobuccal cusp tip and the left deciduous second molar mesiobuccal cusp tip.
7	**Left side angle (degrees)**	The angle between the left deciduous canine cusp tip to the left deciduous second molar mesiobuccal cusp tip and the right deciduous second molar mesiobuccal cusp tip.
8	**Right canine distance to midline (mm)**	Distance between right deciduous maxillary canine cusp tip to midline.
9	**Left canine distance to midline (mm)**	Distance between left deciduous maxillary canine cusp tip to midline.
10	**Right molar distance to midline (mm)**	Distance between right deciduous maxillary second molar mesiobuccal cusp tip to midline.
11	**Left molar distance to midline (mm)**	Distance between left deciduous maxillary second molar mesiobuccal cusp tip to midline.

### Bias

Blinded assessors were trained to place all the landmarks on the digital models. The first assessor was responsible for placing the landmarks on all the models and repeating 20% of the sample after 2 weeks to measure the intra-observer reliability. The second assessor placed the landmarks on the same 20% of the sample to measure the inter-observer reliability.

### Study size

Calculation of the sample size was done using data from a previous study (Bishara et al., 1997) [32], in which the value of the maxillary inter-canine width (ICW) was mentioned for normal non-cleft females at 5 years old of age. This value was 28.4 mm (Standard deviation *D* = 1.7). By setting the power of 80%, type I error of 5%, and using an independent sample *t*-test, effect size of 1.76 resulted. The calculation donated the inclusion of 7 patients in each group. In the current study, this number was increased to avoid any dropouts and to prevent the attrition bias. In the current study, maxillary models of 28 patients were included.

### Statistical methods

The significance level was set at *P* ≤ 0.05. Statistical analysis was performed with IBM® SPSS® Statistics Version 20 for Windows. Handling of data was done using Microsoft Excel software. Inter-class Correlation Coefficient (ICC) was calculated to detect the intra and inter-observer reliability of the selected measurements in the study. The closer the ICC to 1.0, the higher the reliability of the measurement.

Data was explored for normality using Kolmogorov-Smirnov and Shapiro-Wilk tests. According to the behavior of the data (either parametric or nonparametric), a suitable statistical test was selected.

The mean and standard deviation values were calculated for each group in each test. For symmetry measurements (side angles, canine and molar distances to midline), *paired sample t-test was used* to compare the right and left sides. A *one-way ANOVA* test was used to compare between the 3 groups. This was followed by the *Tukey post hoc test* to compare each 2 groups.

## Results

### Participant flow, dropouts, and numbers analyzed

Twenty-eight subjects were included in the current study. Eighteen patients with cleft palate were included and divided into 2 surgical groups: 9 patients in each group. Additionally, 10 non-cleft children were included in the control group. The models of all 28 subjects were analyzed.

### Outcomes and estimation

Intra-observer and inter-observer reliability was assessed between 2 readings done by the 2 assessors to the different measurements using the ICC. Acceptable intra-observer reliability and agreement between all the readings (ICC values ranging from 0.85 to 0.92) were found. For the inter-observer reliability, acceptable reliability was observed for most of the measurements (ICC values ranging from 0.79 to 0.91).

For arch symmetry measurements (side angles, canine, and molar distances to midline), non-significant differences were found between the right and left sides within the 3 groups (Table 2) nor between the 3 groups (Tables 3 and 4).

**Table 2:** Evaluation of arch symmetry; mean and standard deviation of the right and left sides in the 3 groups compared using the paired *t*-test

Measurements	TFP							FPBF							Control						
	Right		Left		Diff		*P*-value	Right		Left		Diff		*P*-value	Right		Left		Diff		*P*-value
	Mean	SD	Mean	SD	Mean diff	SD		Mean	SD	Mean	SD	Mean diff	SD		Mean	SD	Mean	SD	Mean diff	SD	
Side angle (degrees)	59.9	5.8	56.5	8	3.35	5.77	0.119ns	57.1	4.3	52.3	10.4	4.8	11.08	0.230ns	62	2.4	60.7	3.1	1.28	1.75	0.05ns
Canine distance to midline (mm)	13.8	1.1	12.7	1.5	1.05	2.12	0.175ns	13.9	1	12.5	2	1.45	2.5	0.120ns	14.9	1.1	15.1	1.1	0.25	0.54	0.175ns
Molar distance to midline (mm)	20.2	1.3	20.6	1.9	0.36	1.44	0.475ns	20.9	1.2	20.7	1.9	0.2	1.55	0.707ns	21.6	0.8	22.1	1.1	0.48	0.63	0.06ns

*TFP* two flap palatoplasty, *FPBF* Furlow palatoplasty with buccal myomucosal flap, *ns* non-significant (*p*>0.05), *SD* standard deviation

**Table 3:** Mean and standard deviation of the different model measurements by the 3 groups compared using the one-way ANOVA

Measurements	TFP		FPBF		Control		*P*-value
	Mean	SD	Mean	SD	Mean	SD	
Inter-canine width (mm)	26.90^a^	1.81	26.56^a^	2.24	30.18^b^	2.24	0.001*
Intermolar width (mm)	40.74^a^	2.80	41.70 ^a^	2.42	43.17^a^	2.71	0.153ns
Arch length (mm)	23.45 ^a^	1.86	24.09 ^ab^	1.79	26.38 ^b^	1.65	0.003*
Anterior palatal depth (mm)	3.77 ^a^	0.80	4.71 ^a^	0.74	4.58 ^a^	1.93	0.271ns
Posterior palatal depth (mm)	10.74 ^a^	1.62	11.14 ^ab^	1.85	13.16 ^b^	1.84	0.013*
Side angle (degrees)	3.35 ^a^	5.77	4.8 ^a^	11.08	1.28 ^a^	1.75	0.569ns
Canine distance to midline (mm)	1.05 ^a^	2.12	1.45 ^a^	2.5	0.25 ^a^	0.54	0.137ns
Molar distance to midline (mm)	0.36 ^a^	1.44	0.2 ^a^	1.55	0.48 ^a^	0.63	0.508ns

Values for the same letter are not statistically significant, but values having different letters are statistically significant

*TFP* two flap palatoplasty, *FPBF* Furlow palatoplasty with buccal myomucosal flap, *ns* non-significant (*p*>0.05), *SD* standard deviation

*Significant (*p*<0.05)

**Table 4:** Mean differences and 95% confidence interval of the different model measurements between each 2 groups, the *Tukey HSD (Hostely significant difference)* test was used to compare between each 2 groups in each measurement

Measurements	TFP/FPBF			TFP/Control			FPBF/ Control		
	Mean Diff	95% CI	*P*-value	Mean Diff	95% CI	*P*-value	Mean Diff	95% CI	*P*-value
Inter-canine width (mm)	−0.34	−2.81 to 2.14	0.937ns	3.28	0.86 to 5.70	0.006*	3.62	1.19 to 6.03	0.003*
Intermolar width (mm)	0.95	−2.17 to 4.07	0.722ns	2.43	−0.61 to 5.47	0.14ns	1.48	−1.56 to 4.52	0.465ns
Arch length (mm)	0.64	−1.43 to 2.71	0.718ns	2.92	0.90 to 4.94	0.004*	2.29	0.26 to 4.30	0.025ns
Anterior palatal depth (mm)	0.94	−0.59 to 2.48	0.285ns	0.81	−0.69 to 2.31	0.392ns	0.13	−1.63 to 1.37	0.973ns
Posterior palatal depth (mm)	0.40	−1.69 to 2.49	0.879ns	2.42	0.38 to 4.46	0.018*	2.02	−0.01 to 4.06	0.054ns
Side angle (degrees)	1.44	−10.02 to 7.13	0.907ns	2.07	−6.51 to 10.64	0.820ns	3.51	−5.06 to 12.08	0.570ns
Canine distance to midline (mm)	0.40	−2.61 to 1.81	0.892ns	1.31	−0.85 to 3.46	0.310ns	1.71	−0.45 to 3.86	0.145ns
Molar distance to midline (mm)	0.56	−2.09 to 0.97	0.645ns	0.13	−1.45 to 1.70	0.977ns	0.69	−0.89 to 2.27	0.521ns

*TFP* two flap palatoplasty, *FPBF* Furlow palatoplasty with buccal myomucosal flap, *ns* non-significant (*p*>0.05), *CI* confidence interval

*Significant (*p*<0.05)

For the rest of the measurements, significant differences were detected in the inter-canine width, arch length, and posterior palatal depth while comparing the 3 groups (Table 3). After comparing the 2 groups, both TFP and FPBF significantly differ from the control group for the inter-canine width with no difference detected between the 2 surgeries (Table 4). For arch length and posterior palatal depth, significant differences were detected between TFP and the control groups with insignificant differences between the FPBF and control groups (Table 4). Non-significant differences were found for the rest of the measurements between the 3 groups.

## Discussion

Cleft palate repair is associated with the development of palatal scar with its potential impairing effect on the growth of the maxillofacial structures [9, 10]. About 25 to 60% of cleft patients experienced maxillary hypoplasia in transverse, sagittal, and vertical dimensions after cleft repair [9, 10]. Additionally, 70% of the patients have skeletal class 3 associated with the narrow-collapsed maxilla and high vault palate, crossbite, anterior open bite, dental crowding, and mouth breathing which occurs due to scar contracture at the surgical site [11–13].

The selected population in this study were female and their ages were observed between 5 and 6 years old because craniofacial structure attained the adult size at the age of 5.5 years [33–35]. In addition, maxillary growth in female patients was 1–2% more than that in males and this indicates the difference in maturity [33].

All cleft palate in this study was repaired at 9–12 months of age. It is a preferred time for palatoplasty to improve speech development. But it was found that early palatoplasty affects the growth of the dental arch and maxilla [36].

Various surgical techniques are followed for cleft palate repair but TFP is considered as the most commonly used technique [19–21]. It is used to close the hard and soft palate in one operation without tension and provides proper reorientation of soft palate musculature [37]. In addition, it is associated with a low fistula rate and less impact on maxillary growth attributed to less hard palate bone exposure and mucoperiosteum elevation [22–25]. In contrast, Koberg and Koblin [38] approved that TFP and Veau’s technique were the most harmful techniques with a restricted effect on maxillary growth. This also was supported by Mann et al. [26] who found that maxillary growth restriction is due to scar formation of two flap technique relaxing incision. Furlow palatoplasty with buccal myomucosal flap was introduced to the surgical theater by Mann RJ et al. [26] in 2017 as a modification of Furlow’s Z-plasty technique for surgical repair of cleft palate. Furlow palatoplasty with buccal myomucosal flap combines the advantages of using Furlow opposing Z flap and buccal myomucosal flap. It is associated with a low fistula rate due to tensionless flap closure, increases the soft palate length that improves the speech quality in cleft patients, and removes the need for relaxing incision that causes scare formation and impairs the maxillary growth [21, 26]. We believe that the structural defect and tissue deficiency in cleft palate need to be reconstructed by a smiling soft pliable tissue as the buccinator myomucosal flap, which in turn would allow for normal midfacial growth.

Despite the undetected differences between the 3 groups for the arch symmetry measurements, differences were obvious among the other arch measurements. For the FPBF and in comparison to the control group, no significant differences were detected in all the measurements except for the inter-canine width. Most probably the Z style of flap design and closure and the integration of the buccal myomucosal flaps delivered a maxillary arch with closer dimensions to the studied normal subjects.

On the contrary, more differences were detected between the TFP and normal control subjects. After observing the differences between the 2 groups, significant differences were found, not only in the inter-canine width but also in the arch length and the posterior palatal depth. This might be due to the straight-line closure of the two flaps and its anteroposterior scar contraction, in addition to the absence of the extra augmentation of the buccal myomucosal flap presented in the FPBF group. In the current study, TFP technique was followed without any augmentation from the buccal tissues, neither the buccal myomucosal flap nor the buccal pad fat which might be the reason for the observed dentoalveolar changes that occurred in this group. The use of a pedicel of buccal pad fat with TFP might be with more advantages but further studies are needed to confirm its effectiveness [39].

One of the limitations of the current study was the wide range of age (9–12 years) at which the surgeries were done. The study design is considered as another limitation of this study because the cohort study design is with some inherent biases like selection bias. This study compared the 2 clefted groups with a normal population. Accordingly, it could not be performed in a randomized controlled trial (RCT) design. Despite the limitation of the cohort study, the performance of sample size calculation is considered a point of strength. Another point of strength is the use of digital models for the evaluation of the maxillary arch dimensions between the 3 groups. Digital models are more versatile while evaluating the dental arches in the 3 dimensions especially with the vertical dimension evaluation. Also, all the surgeries were done by a single experienced plastic surgeon, and this is considered a point of strength.

The results of the current study are of clinical value and might affect the decision of choosing the flap design for cleft palate repair. It seems that the flap design, the style of closure, and the incorporation of the buccal myomucosal flap affect the maxillary arch shape at the age of 5 years in patients with cleft palate.

## Conclusions

Within the limitations of the current study, the following can be concluded:

1. Furlow palatoplasty with buccal myomucosal flap resulted in better maxillary arch dimensions in comparison to two flap palatoplasty in patients with cleft palate at the primary dentition stage.
2. Both Furlow palatoplasty with buccal myomucosal flap and two flap palatoplasty produced symmetric maxillary arches in comparison to the non-cleft subjects.
3. Furlow palatoplasty with buccal myomucosal flap might be the design of choice while treating patients with cleft palate.

## Data Availability

The study’s datasets are not publicly available for data protection and security but can be obtained from the corresponding author on reasonable consideration.

## Abbreviations

FPBF: Furlow Palatoplasty with Buccal Myomucosal flap
ICC: Inter-class correlation
ICW: inter-canine width
RCT: randomized controlled trial
STROBE: Strengthening the Reporting of Observational studies in Epidemiology
TFP: two flap palatoplasty
