# Correction to: Effects of two fap palatoplasty versus furlow palatoplasty with buccal myomucosal fap on maxillary arch dimensions in patients with cleft palate at the primary dentition stage: a cohort study

**DOI:** 10.1007/s00784-023-05200-1

**Published:** 2023-08-12

**Authors:** Mamdouh Ahmed Aboulhassan, Shaimaa Mohsen Refahee, Shaimaa Sabry, Mohamed Abd‑El‑Ghafour

**Affiliations:** 1 Plastic Section, Department of General Surgery, Faculty of Medicine, Cairo University, Cairo, 11111 Egypt; 2 Oral and Maxillofacial Surgery Department, Faculty of Dentistry, Fayoum University, Fayoum, 63511 Egypt; 3 Department of Pediatric Dentistry and Public Health, Faculty of Dentistry, Cairo University, Cairo, 11111 Egypt; 4 Department of Orthodontics, Faculty of Dentistry, Cairo University, Cairo, 11111 Egypt

**Correction to: Clinical Oral Investigations**

https://doi.org/10.1007/s00784-023-05182-0

In Fig. 3, images (c) and (d) were repeated as a copy of image (b). In the corrected Fig. 3, the correct images number (c) and (d) were added with marks of measurements from number 6 to number 11 matching the following figure legend.

Figure 3: a Model’s measurements; 1- Inter-canine width (mm), 2- Intermolar width (mm), 3- Arch Length (mm), b Model’s measurments;4- Anterior Palatal Depth (mm), 5-Posterior Palatal Depth (mm), c Model’s measurements; 6- Right Side Angle (degrees), 7- Left Side Angle (degrees), d Model’s measurements; 8- Right Canine Distance to midline (mm), 9- Left Canine Distance to midline (mm), 10- Right Molar Distance to midline (mm), 11- Left Molar Distance to midline (mm).

The original article has been corrected.

